# Comparative field efficacy of all-in-one intradermal vaccine based on an inactivated recombinant *Mycoplasma hyopneumoniae* with an embedded/integrated porcine circovirus type 2 (PCV2) capsid protein and concurrent administration with monovalent of *M. hyopneumoniae* and PCV2 intradermal vaccine

**DOI:** 10.3389/fvets.2025.1428263

**Published:** 2025-03-26

**Authors:** Jeongmin Suh, Sehyeong Ham, Chanhee Chae

**Affiliations:** Department of Veterinary Pathology, College of Veterinary Medicine, Seoul National University, Seoul, Republic of Korea

**Keywords:** intradermal vaccine, enzootic pneumonia, *Mycoplasma hyopneumoniae*, porcine circovirus type 2, porcine respiratory disease complex

## Abstract

**Background:**

Efficacy of an intradermal all-in-one vaccine based on an inactivated recombinant *Mycoplasma hyopneumoniae* strain with an embedded/integrated porcine circovirus type 2 (PCV2) capsid protein thereof, as the single active substance, is provided.

**Objective:**

To evaluate and compare an intradermal *M. hyopneumoniae* and PCV2 all-in-one vaccine with monovalent of *M. hyopneumoniae* and PCV2 intradermal vaccines administered concurrently.

**Animals:**

A total of 60 pigs were randomly divided into three groups (20 pigs per group; male =10 and female = 10) in each of three farms.

**Methods:**

The clinical field trials were conducted on three farms, each suffering from PCV2 infection and enzootic pneumonia. The pigs in the all-in-one-vaccinated groups were injected intradermally with 0.2 mL of the all-in-one vaccine through a needle-free device. The pigs in the concurrent-vaccinated groups were injected with 0.2 mL of PCV2 vaccine and 0.2 mL of *M. hyopneumoniae* vaccine intradermally through a needle-free device, which allows two vaccines each in their separate vials to be applied simultaneously. The pigs in the unvaccinated groups were administered an equal volume of phosphate buffered saline in the same manner at 21 days of age.

**Results:**

Intradermal vaccination improved pig production performance. It was also able to stimulate systemic humoral and cell-mediated immune responses to *M. hyopneumoniae* and PCV2d, which reduced *M. hyopneumoniae* nasal loads and the severity of mycoplasmal lung lesions. Through this stimulation, PCV2 blood viral load and lymphoid severity were also reduced.

**Discussion:**

The intradermal vaccines were considered efficacious and conferred cross-protection of pig herds suffering from PCV2 infection and enzootic pneumonia.

## Introduction

Porcine circovirus type 2 (PCV2) is the primary causative pathogen of porcine circovirus-associated disease (PCVAD), which collectively represents the many clinical manifestations of PCV2 infections such as postweaning multisystemic wasting syndrome (PMWS), porcine respiratory disease complex (PRDC), reproductive disorders, and enteric diseases (1–3). Subclinical PCV2 infection is currently considered the most common form of PCVAD due to the widespread use of efficacious PCV2 vaccine (3). Similarly, the worldwide spread of enzootic pneumonia is often caused by *Mycoplasma hyopneumoniae*, which frequently leads to subclinical pig infection. This is categorized by a chronic dry cough, unthrifty pig appearance, and reduced pig growth (4, 5).

Pig coinfection of pigs with PCV2 and *M. hyopneumoniae* is recognized as part of PRDC, which is responsible for enormous economic losses to global pig industry (6, 7). Until the recent introduction and gain in popularity of intradermal vaccines, traditional, intramuscularly-administered bivalent vaccines have been the most commonly used worldwide method of controlling PCV2 and *M. hyopneumoniae* coinfections in pigs. Intradermal vaccination can prime a strong humoral and cellular immune response comparable to that obtained by intramuscular vaccination (8). Additional advantages in using a needle-free intradermal route of antigen administration are pig pain and stress minimization, and an improvement in meat quality due to the lack of needle-induced injection site lesions and the removal of needle breakage risk. The development and usage of intradermal vaccines is likely to continue growing within the veterinary field due to these apparent advantages.

Although commercially available intradermal monovalent vaccines are used to control either *M. hyopneumoniae* or PCV2 infection (9, 10), an intradermal bivalent vaccine used to control both pathogens is not yet commercially available. This study evaluated an all-in-one vaccine (MHYOSPHERE^®^ PCV ID, Laboratorios HIPRA S.A., Amer, Spain) that is based on an inactivated recombinant *M. hyopneumoniae* strain called Nexhyon with an embedded/integrated PCV2 capsid protein thereof, as the single active substance. MHYOSPHERE^®^ PCV ID was explicitly developed for intradermal administration using a needle free and intradermal injector (11). An all-in-one vaccine (MHYOSPHERE^®^ PCV ID, Laboratorios HIPRA S.A.) conferred protection against co-challenge of *M. hyopneumoniae* and PCV2d under experimental conditions (11). Therefore, the objective of this study was to determine and compare the efficacy of a new intradermal vaccine of *M. hyopneumoniae* and PCV2 (hereafter called the “all-in-one vaccine”) with the intradermal monovalent vaccines of *M. hyopneumoniae* and PCV2 administered through a needle-free device which allows two monovalent vaccines in two separate vials to be applied simultaneously (hereafter called the “concurrent vaccine”) under field conditions.

## Materials and methods

### Ethical statement

All of the methods were approved by the Seoul National University Institutional Animal Care and Use, and Ethics Committee (SNU-220117-1).

### Farm history

The clinical field trial was conducted on three farms. Farms were labeled as “A, B, and C” and were 410-sow, 210-sow, and 320-sow (respectively) farrow-to-finish swine operations with an all-in-all-out production system. Sows from the three farms were not immunized for either PCV2 or *M. hyopneumoniae*. All piglets received vaccinations for PCV2 and *M. hyopneumoniae* at 3 weeks of age. The status of porcine reproductive and respiratory syndrome (PRRS) was stable, with no active PRRSV circulation (high-parity sows are the only seropositive animals in the herd) on three farms.

Farms A, B, and C were selected based on their history of subclinical PCV2 infection and *M. hyopneumoniae* infection. Each farm consistently suffered pig loss over several months due to growth retardation and respiratory disease during the late post-weaning and growing stages. Previous diagnoses fulfilled the definition of subclinical PCV2 infection (3) to include decreased average daily gain without overt clinical signs, absence of or minimal histopathological lesions in superficial inguinal lymph nodes, and a low amount of PCV2 antigen presence in superficial inguinal lymph nodes as determined by immunohistochemistry in two out of three suspected pigs from the three farms. PCV2d was detected in serum from three pigs with each of these three farms, where log10 DNA copies/mL ranged from 5.99 to 6.72 from farm A, 4.77 to 6.66 from farm B, and 6.02 to 7.03 from farm C by real-time PCR DNA in two out of three pigs at the 8 weeks of age from farm A, in two out of two pigs at 9 weeks of age from farm B, and two out of four pigs at the 8 weeks of age from farm C. In addition, a histological lung examination was performed at the same age, which confirmed that two out of three pigs from farm A, one out of two pigs from farm B, and two out of four pigs from farm C had mycoplasma pneumonia lesions.

### Clinical field design

The experimental design for the field study strictly adhered to the guidelines set by the Republic of Korea’s Animal, Plant & Fisheries Quarantine & Inspection Agency (QIA, http://www.qia.go.kr). To minimize sow variation, six 18-day-old piglets (three males and three females, of similar weight) were randomly selected using the random number generator function (Excel, Microsoft Corporation, Redmond, WA, United States) from each sow and assigned evenly (one male and one female per sow) to each of the three groups in each of three farms. A total of 60 pigs were randomly divided into three groups (20 pigs per group; male =10 and female = 10) using the same software and function in each of three farms. Pigs were identified by ear notching.

At 0 days post-vaccination (dpv, 21 days of age), the pigs in the VacA1, VacB1, and VacC1 groups were each administered 0.2 mL of *M. hyopneumoniae* and PCV2 all-in-one vaccine (MHYOSPHERE^®^ PCV ID, Laboratorios HIPRA S.A. antigen content of *M. hyopneumoniae*: relative potency ≥1.3, antigen content of PCV2: relative potency ≥1.3) through the intradermal route through a needle-free device (Hipradermic^®^, Laboratorios HIPRA S.A.). Each farm received a different vaccine serial as follows: Farm A = Batch No. I0EH5 (Expiration date Apr-2025), Farm B = Batch No. I0EH6 (Expiration date Apr-2025), and Farm C = Batch No. I0EH7 (Expiration date Apr-2025). The pigs in the VacA2, VacB2, and VacC2 groups received 0.2 mL of monovalent PCV2 vaccine (Porcilis^®^ PCV ID, antigen content of PCV2: antigen unit ≥1,436, Lot No. A079A01, Expiration date: May-2024, MSD Animal Health, Boxmeer, Netherlands) and 0.2 mL of monovalent *M. hyopneumoniae* vaccine (Porcilis^®^ M HYO ID ONCE, antigen content of *M. hyopneumoniae*: packed cell volume units inducing ≥6.5 log_2_ antibody titer, Lot No. A094B01, Expiration date: 29-Aug-2024, MSD Animal Health) through the intradermal route with a needle-free device (twin-nozzle IDAL^®^ 3G Twin, Henke-Sass Wolf, Germany) which allows two vaccines in two separate vials to be applied simultaneously as concurrent vaccine. The pigs in the UnVacA, UnVacB, and UnVacC groups were administered an equal volume of phosphate buffered saline (PBS, 0.01 M, pH 7.4, 0.2 mL) through the intradermal route through a needle-free device (Hipradermic^®^, Laboratorios HIPRA S.A.). Pigs were allowed to commingle between treatments to minimize pen variation. They were randomly reassigned into six pens (10 pigs per pen) within the same building. All animals were housed within the same building in similar conditions, received the same feed, and were subjected to the same management practices.

Five pigs from each group were randomly selected and euthanized for necropsy at 91 days post-vaccination (dpv) (112 days of age). The rest of the pigs from each group were euthanized for necropsy at 154 dpv (175 days of age). Pigs were sedated by an intravenous injection of sodium pentobarbital and then euthanized as previously described (12). Tissues such as lung and subinguinal lymph node were collected from each pig at necropsy and fixed for 24 h in 10% neutral buffered formalin, routinely processed, and embedded in paraffin.

### Sampling collection

Blood, and fecal and nasal swabs will be collected from all pigs at 0 (21 days of age), 28 (49 days of age), 49 (70 days of age), 91 (112 days of age), and 154 (175 days of age) dpv.

### Clinical observation

Pigs were monitored daily and scored weekly for clinical signs. Scoring was defined according to the following scale: 0 (normal), 1 (rough haircoat), 2 (rough haircoat and mild dyspnea), 3 (mild dyspnea and abdominal breathing), 4 (moderate dyspnea and abdominal breathing), 5 (severe dyspnea and abdominal breathing), and 6 (death). Scoring observers were blinded to vaccination status.

### Growth performance

The live weight of each pig was measured at 0 (21 days of age), 49 (70 days of age), 91 (112 days of age), and 154 (175 days of age) dpv. The average daily weight gain (ADWG; gram/pig/day) was analyzed over three time periods: (i) between 0 and 49 dpv, (ii) between 49 and 91 dpv, (iii) between 91 and 154 dpv, and (iv) between 0 and 154 dpv. ADWG during the different production stages was calculated as the difference between the starting and final weight divided by the duration of the stage. Data for dead or removed pigs were included in the calculation.

### Mortality

During the field trials, dead pigs were submitted to the diagnostic laboratory at Seoul National University. Diagnosis was carried out using routine methods, as previously described (13).

### Quantification of PCV2d DNA in serum, nasal and fecal samples

DNA was extracted from serum, and nasal and fecal samples using the commercial kit (QIAamp DNA Mini Kit, QIAGEN, Valencia, CA) to quantify PCV2d genomic DNA copy numbers by real-time PCR. The assay’s detection limit was 1.2 × 10^2^ genomic copy numbers of PCV2d (14, 15).

### Quantification of *Mycoplasma hyopneumoniae* DNA in laryngeal samples

DNA was extracted from laryngeal swabs using the commercial kit (QIAamp DNA Mini Kit, QIAGEN) to quantify the *M. hyopneumoniae* genomic DNA copy numbers by real-time PCR (16). The assay’s detection limit was 1.3 × 10^2^ genomic copy numbers of *M. hyopneumoniae* (16, 17).

### Serology

The presence of PCV2 and *M. hyopneumoniae* antibodies was evaluated in serum samples by use of commercially available enzyme-linked immunosorbent assay (ELISA) kits (Porcine Circovirus type 2 Antibody Test, BioChek B.V., Reeuwijk, Holland and *M. hyo* Ab test, IDEXX Laboratories Inc. Westbrook, Maine, United States) according to the manufacturer’s instructions. Serum samples were considered positive for the presence of PCV2 antibody when the average sample-to-positive ratio (S/P) ratio was ≥0.5 and as positive for *M. hyopneumoniae* antibody if the S/P ratio was ≥0.4. The serum viral neutralization test was performed in 96-well microtitration plates using PK-15 cells as the indicator (14, 18, 19).

### Enzyme-linked immunospot

An enzyme-linked immunospot (ELISpot) assay was conducted to measure the numbers of PCV2d- and *M. hyopneumoniae*-specific interferon-γ secreting cells (IFN-γ-SC). Peripheral blood mononuclear cells (PBMC) were stimulated using the aforementioned challenge strains for PCV2d and *M. hyopneumoniae* with results reported as the number of IFN-γ-SC per million PBMC (14, 20).

### Pathology

Two pathologists at the Seoul National University scored the severity of macroscopic lung lesions blindly in order to estimate the percentage of the lung affected by pneumonia (21). Two blinded veterinary pathologists then examined the collected lung and lymphoid tissue sections. The severity of lung lesions was evaluated for presence of peribronchial and peribronchiolar lymphoid hyperplasia, and the amount of inflammation in the lamina propria of bronchi and bronchioles ranging from 0 to 6 (0 = normal; 1 = mild multifocal; 2 = mild diffuse; 3 = moderate multifocal; 4 = moderate diffuse; 5 = severe multifocal; 6 = severe diffuse) (22). The severity of lymphoid lesions was evaluated for presence of lymphoid depletion and inflammation, and given a score ranging from 0 to 5 (0 = normal; 1 = mild lymphoid depletion; 2 = mild to moderate lymphoid depletion and histiocytic replacement; 3 = moderate diffuse lymphoid depletion and histiocytic replacement; 4 = moderate to severe lymphoid depletion and histiocytic replacement; 5 = severe lymphoid depletion and histiocytic replacement) (23).

### Immunohistochemistry

Immunohistochemistry for PCV2 was performed as previously described (24). Three sections were cut from each of three blocks of tissue from a lymph node of each pig and prepared on slides for the morphometric analyses of immunohistochemistry. Quantitative data were analyzed from the prepared immunohistochemistry slides using the NIH ImageJ 1.45s Program.^1^ PCV2 analysis was conducted by the random selection of 10 fields, where number of the positive cells per unit area (0.25 mm^2^) was determined as previously described (25). The mean values were also calculated.

### Statistical analysis

For statistical processing, real-time PCR data and neutralizing antibody titers were converted into decimal logarithmic and binary logarithmic values, respectively. A normal distribution was determined with the Shapiro–Wilk on these data. Whether or not the groups had statistically significant differences between them at various timepoints was then determined by performing a one-way ANOVA. For further evaluation, a post-hoc test for a pairwise comparison with Tukey’s adjustment was conducted with a statistical significance result from a one-way ANOVA test. A Kruskal–Wallis test was additionally performed only in cases where the normality assumption was not met. Results which showed statistical significance from the Kruskal–Wallis test were further evaluated with the Mann-Whitney *U* test to compare the differences among the groups. Results were reported in *p*-values and the values of *p* < 0.05 were considered significant.

## Results

### Clinical signs

**Figure 1:**
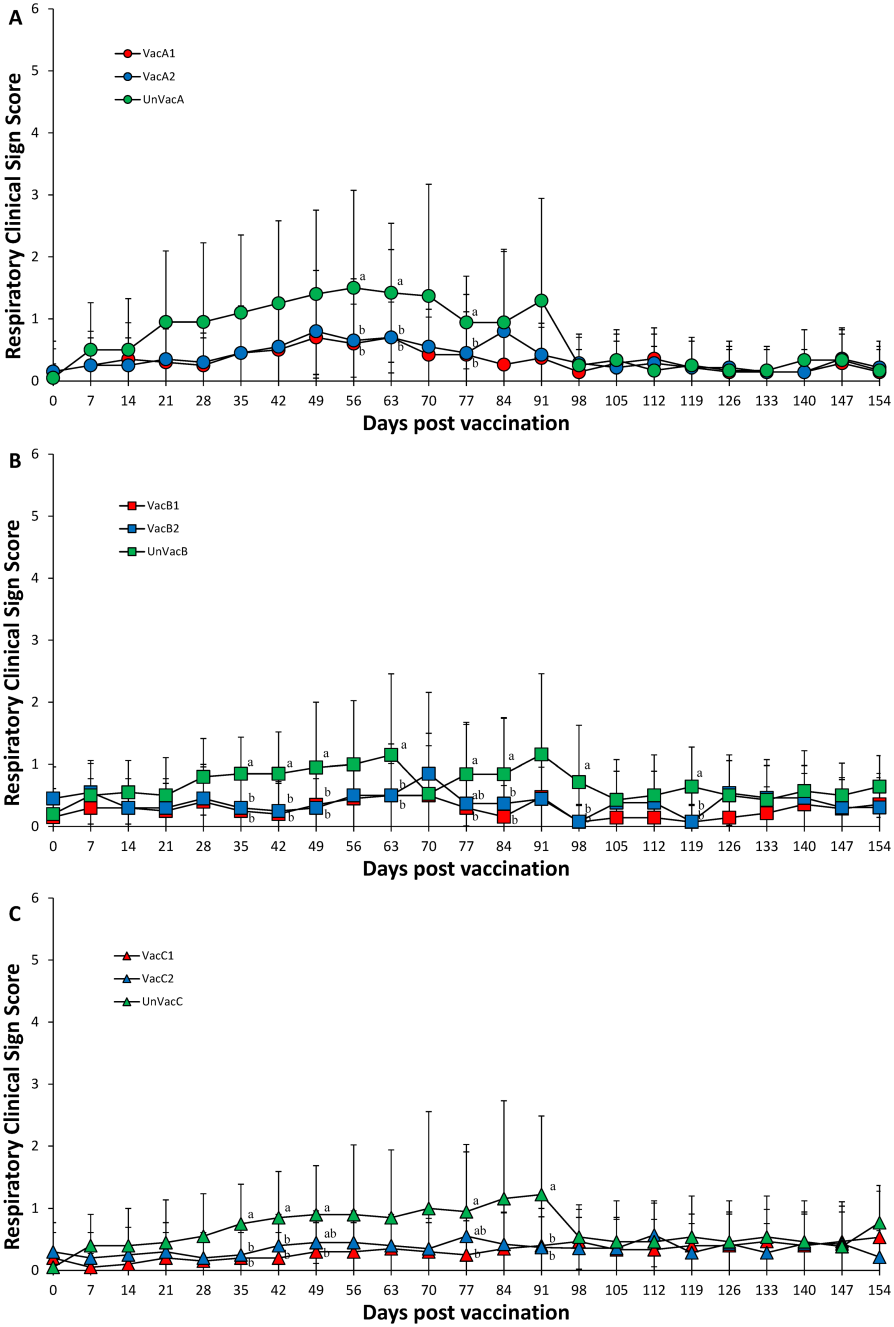
Respiratory clinical scores. **(A)** Mean values of respiratory clinical scores in the farm A. **(B)** Mean values of respiratory clinical scores in the farm B. **(C)** Mean values of respiratory clinical scores in the farm C. Variation is expressed as the standard deviation. Different letters within a sampling point mean statistically significant differences (*p* < 0.05).

In farm A, two vaccinated groups had significantly (*p* < 0.05) lower respiratory sign scores than those in the unvaccinated group at 56, 63, and 77 dpv (Figure 1A). In farm B, two vaccinated groups had significantly (*p* < 0.05) lower respiratory sign scores than those in the unvaccinated group at 35, 42, 49, 63, 77, 84, 98, and 119 dpv (Figure 1B). In farm C, two vaccinated groups had significantly (*p* < 0.05) lower respiratory sign scores than those in unvaccinated group at 35, 42, and 91 dpv. The all-in-one-vaccinated group had significantly (*p* < 0.05) lower respiratory sign scores than those in unvaccinated group at 49 and 77 dpv (Figure 1C). No statistical difference occurred between the two vaccinated groups at all three farms.

### Mortality

One all-in-one-vaccinated pig in the VacA1 group died from streptococcal meningitis at 87 days old. One concurrent-vaccinated pig in the VacA2 group died of bronchopneumonia from a coinfection of *M. hyopneumoniae* (PCR positive) and *Pasteurella multocida* (isolation) at 109 days old. Three unvaccinated pigs in the UnVacA group died from bronchopneumonia by coinfection of *M. hyopneumoniae* (PCR positive) and *P. multocida* (isolation) at 80 days old (one pig) and *M. hyopneumoniae* (PCR positive) and *Trueperella pyogenes* (isolation) at 93 days old (two pigs).

One all-in-one-vaccinated pig in the VacB1 group died from polyserositis by infection of *Glaesserella parasuis* (isolation) at 101 days old. Two concurrent-vaccinated pigs from the VacB2 group died of bronchopneumonia by coinfection of PCV2d (PCR positive), *M. hyopneumoniae* (PCR positive), and *Staphylococcus aureus* (isolation) at 95 days old, and from polyserositis by a coinfection of PCV2d (PCR positive) and *G. parasuis* (isolation) at 108 days old, respectively. One unvaccinated pig in the UnVacB group died from bronchopneumonia caused by a PCV2d (PCR positive), *M. hyopneumoniae* (PCR positive), and *T. pyogenes* (isolation) coinfection at 86 days old.

One concurrent-vaccinated pig in the VacC2 group died from hemorrhagic enteritis with unknown etiology at 104 days old. One unvaccinated pig in the UnVacC group died from bronchopneumonia resulting from a coinfection of PCV2d (PCR positive) and *P. multocida* (isolation) at 87 days old and unknown etiology at 108 days old.

### Growth production

**Figure 2:**
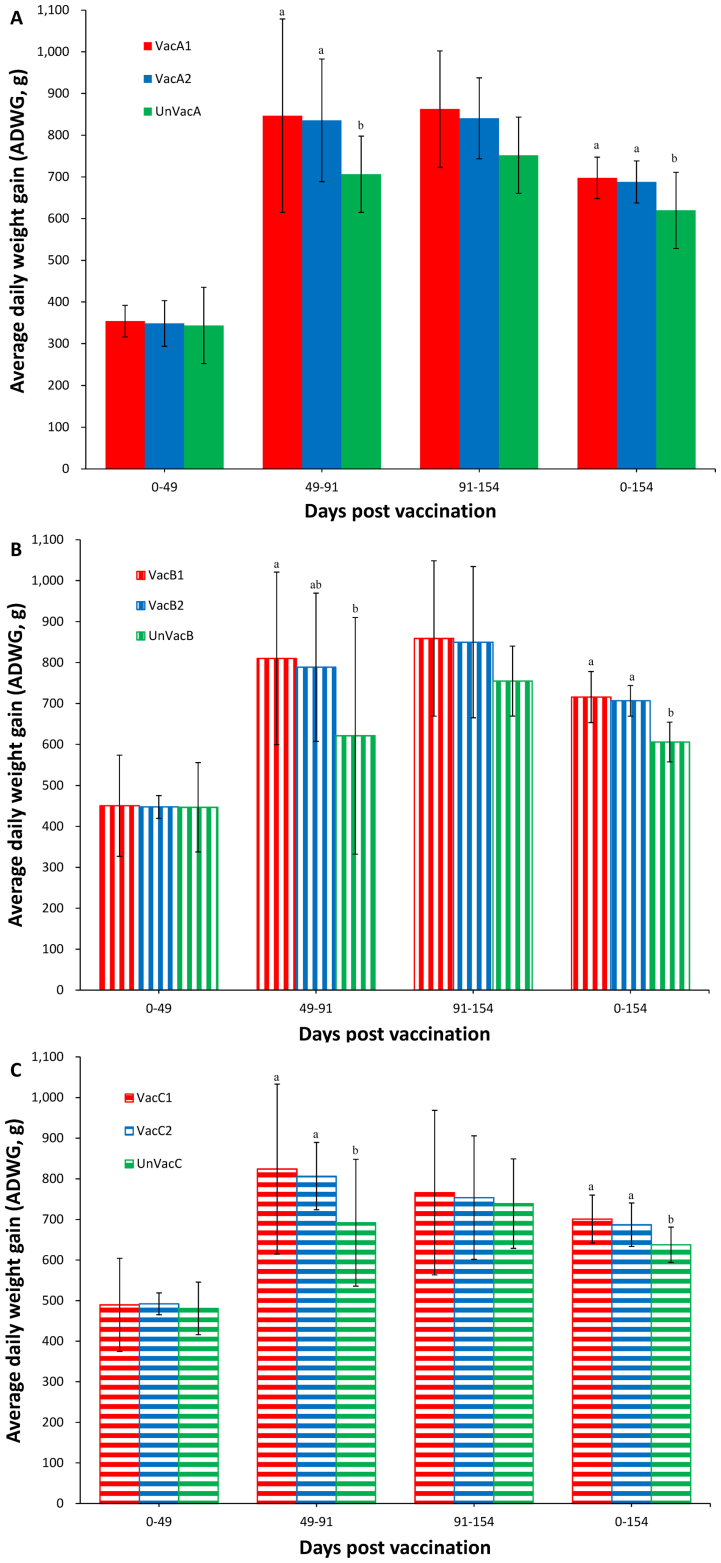
Average daily weight gain (ADWG). **(A)** Mean values of ADWG in the farm A. **(B)** Mean values of ADWG in the farm B. **(C)** Mean values of ADWG in the farm C. Variation is expressed as the standard deviation. Different letters within a sampling point mean statistically significant differences (*p* < 0.05).

Although body weights were not significantly different at the beginning of the study (0 dpv, 21 days of age), statistical difference was measured at 91 (112 days of age) and 154 (175 days of age) dpv between the two vaccinated and the unvaccinated group in three farms. In farms, A and C, two vaccinated groups had a significantly (*p* < 0.05) higher ADWG than that of the unvaccinated group throughout the fattening period (49 to 91 dpv, 70 to 112 days of age) and overall period (0 to 154 dpv, 21 to 175 days) (Figures 2A,C). In farm B, the all-in-one-vaccinated group had a significantly (*p* < 0.05) higher ADWG than that of the unvaccinated group throughout the fattening period (49 to 91 dpv, 70 to 112 days of age). Two vaccinated groups had a significantly (*p* < 0.05) higher ADWG than that of the unvaccinated pigs during the overall period (0 to 154 dpv, 21 to 175 days of age) (Figure 2B).

### PCV2d DNA in serum samples

**Figure 3:**
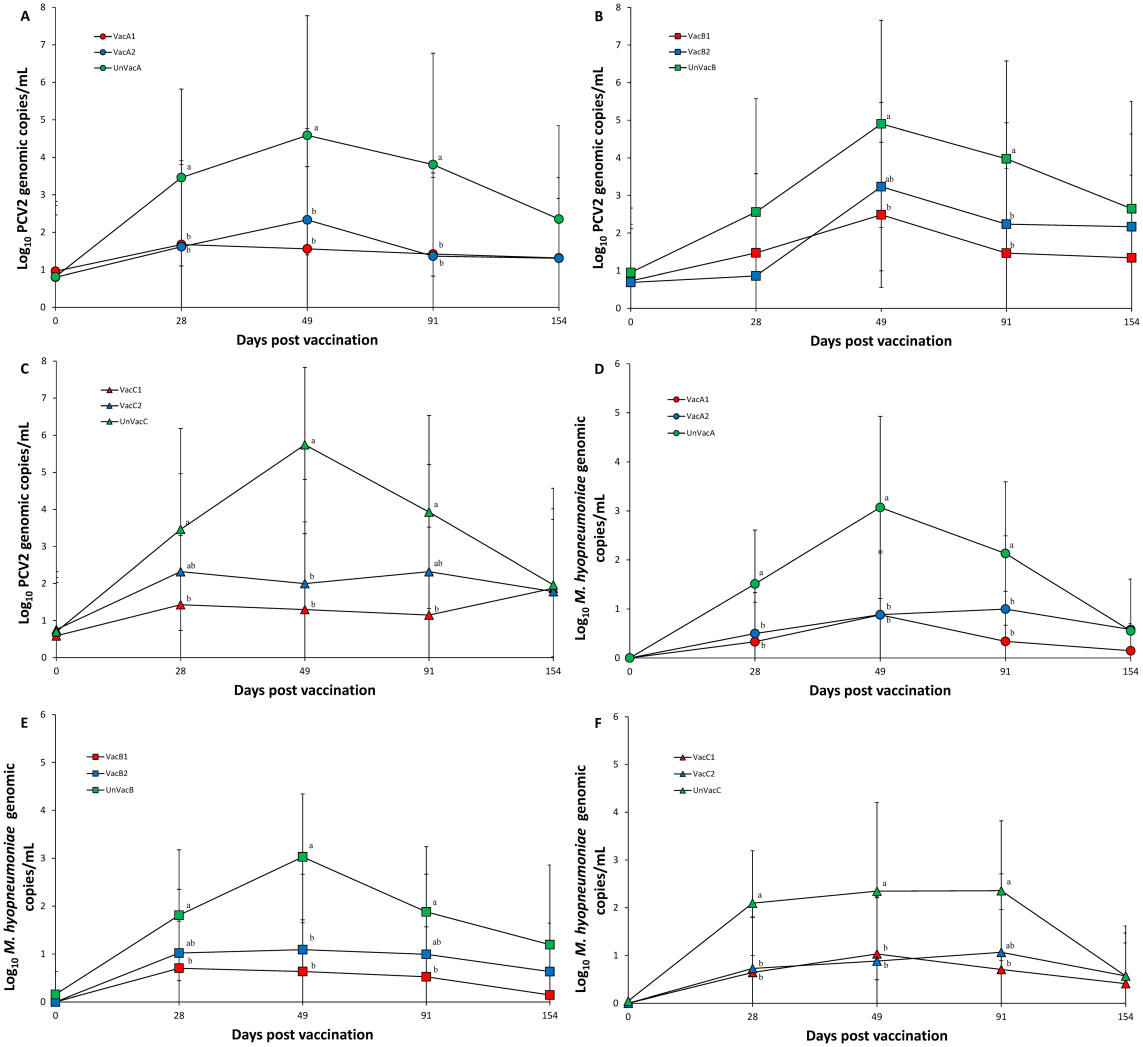
Genomic copies of PCV2d DNA in serum and *M. hyopneumoniae* in nasal samples. **(A)** Mean values of the genomic copies of PCV2d DNA in the farm A. **(B)** Mean values of the genomic copies of PCV2d DNA farm B. **(C)** Mean values of the genomic copies of PCV2d DNA in the farm C. **(D)** Mean values of the genomic copies of *M. hyopneumoniae* DNA in the farm A. **(E)** Mean values of the genomic copies of *M. hyopneumoniae* DNA in the farm B. **(F)** Mean values of the genomic copies of *M. hyopneumoniae* DNA in the farm C. Variation is expressed as the standard deviation. Different letters within a sampling point mean statistically significant differences (*p* < 0.05).

Vaccination of two groups within farm A significantly (*p* < 0.05) lowered the amount of measured PCV2d genomic copies in the serum samples compared to the unvaccinated group at 28, 49, and 91 dpv (Figure 3A). Within farm B, the all-in-one-vaccinated group resulted in a significantly (*p* < 0.05) lower amount of PCV2d genomic copies as measured from serum samples than those of the unvaccinated group at 49 and 91 dpv. The concurrent-vaccinated group significantly (*p* < 0.05) lowered the amount of PCV2d genomic copies in the serum samples compared to the unvaccinated group at 91 dpv (Figure 3B). In farm C, the all-in-one-vaccinated group resulted in a significantly (*p* < 0.05) lower amount of PCV2d genomic copies in the serum samples than those from the unvaccinated group at 28, 49, and 91 dpv. The concurrent-vaccinated group significantly (*p* < 0.05) lowered the amount of PCV2d genomic copies in the serum samples compared to the unvaccinated group at 49 dpv (Figure 3C).

### *Mycoplasma hyopneumoniae* DNA in laryngeal samples

Vaccination of two groups in both farms, A and C, significantly (*p* < 0.05) lowered the amount of *M. hyopneumoniae* genomic copies in the laryngeal samples compared to the unvaccinated group at 28 and 49 dpv. All-in-one vaccination resulted in a significantly (*p* < 0.05) reduced amount of *M. hyopneumoniae* genomic copies in laryngeal samples compared to the unvaccinated group at 91 dpv (Figures 3D,F). Within farm B, all-in-one-vaccination significantly (*p* < 0.05) reduced the amount of *M. hyopneumoniae* genomic copies in the laryngeal samples compared to the unvaccinated group at 28 and 91 dpv. Vaccination of both groups significantly (*p* < 0.05) reduced the amount of *M. hyopneumoniae* genomic copies in the laryngeal samples compared to the unvaccinated group at 49 dpv (Figure 3E).

### Immune responses against PCV2

**Figure 4:**
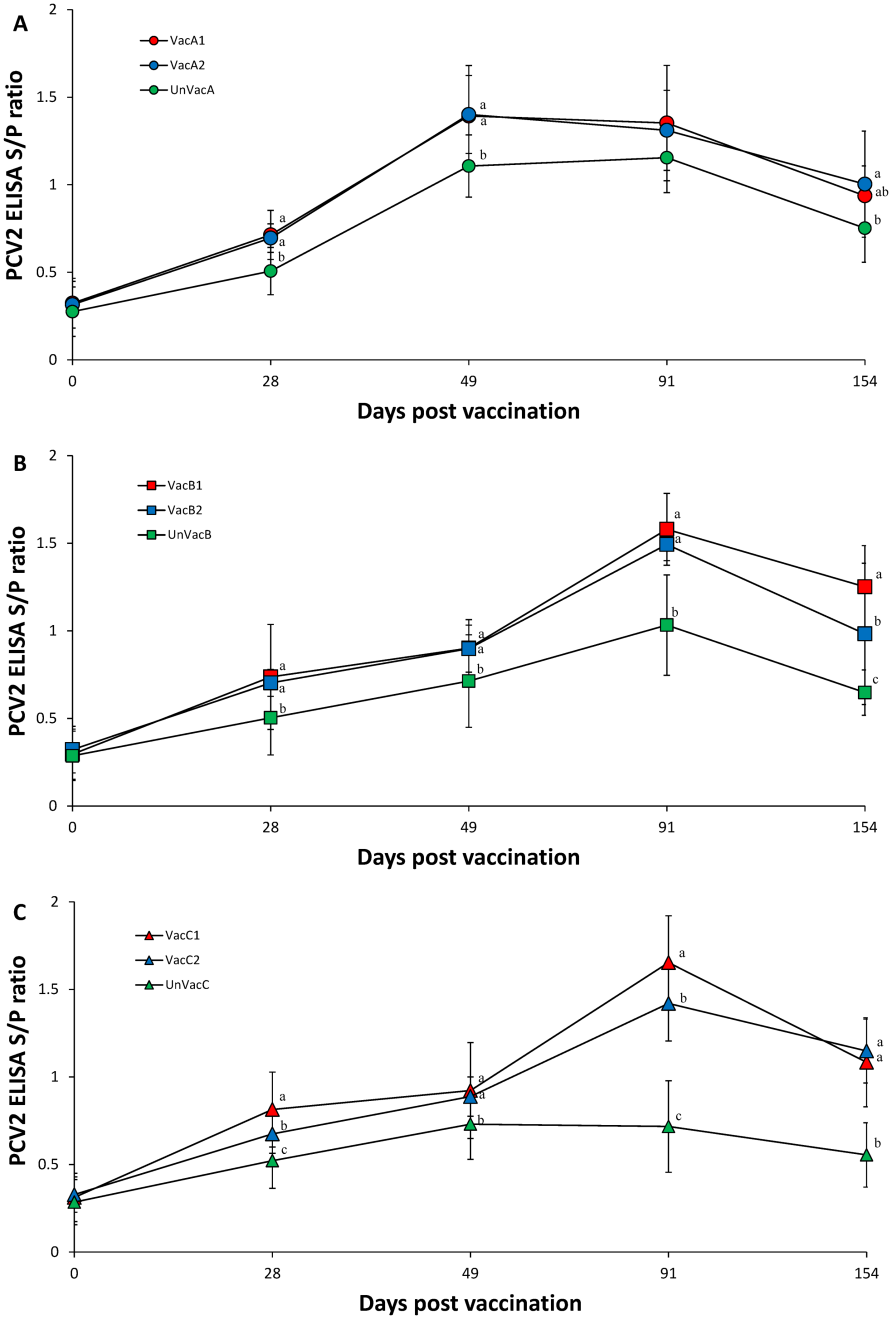
Porcine circovirus type 2 (PCV2) antibodies. **(A)** Mean values of the ELISA PCV2 antibodies in the farm A. **(B)** Mean values of the ELISA PCV2 antibodies in the farm B. **(C)** Mean values of the ELISA PCV2 antibodies in the farm C. Variation is expressed as the standard deviation. Different letters within a sampling point mean statistically significant differences (*p* < 0.05).

Vaccination of two groups in farm A (VacA1 and VacA2) yielded significantly (*p* < 0.05) higher PCV2 S/P ratios compared to the unvaccinated group (UnVacA) at 28 and 49 dpv. The concurrent-vaccinated group (VacA2) had significantly (*p* < 0.05) higher PCV2 S/P ratios compared to the unvaccinated group (UnVacA) at 154 dpv (Figure 4A). Vaccination of two groups in farm B yielded significantly (*p* < 0.05) higher PCV2 S/P ratios compared to the unvaccinated group (UnVacB) at 28, 49, 91, and 154 dpv. The all-in-one-vaccinated group (VacB1) yielded significantly (*p* < 0.05) higher PCV2 S/P ratios than that of the concurrent-vaccinated group (VacB2) at 154 dpv (Figure 4B). Within farm C, the two vaccinated groups (VacC1 and VacC2) had significantly (*p* < 0.05) higher PCV2 S/P ratios than that of the unvaccinated group (UnVacC) at 28, 49, 91, and 154 dpv. The all-in-one-vaccinated group (VacC1) yielded a significantly (*p* < 0.05) higher PCV2 S/P ratios compared to the concurrent-vaccinated group (VacC2) at 28 and 91 dpv (Figure 4C).

**Figure 5:**
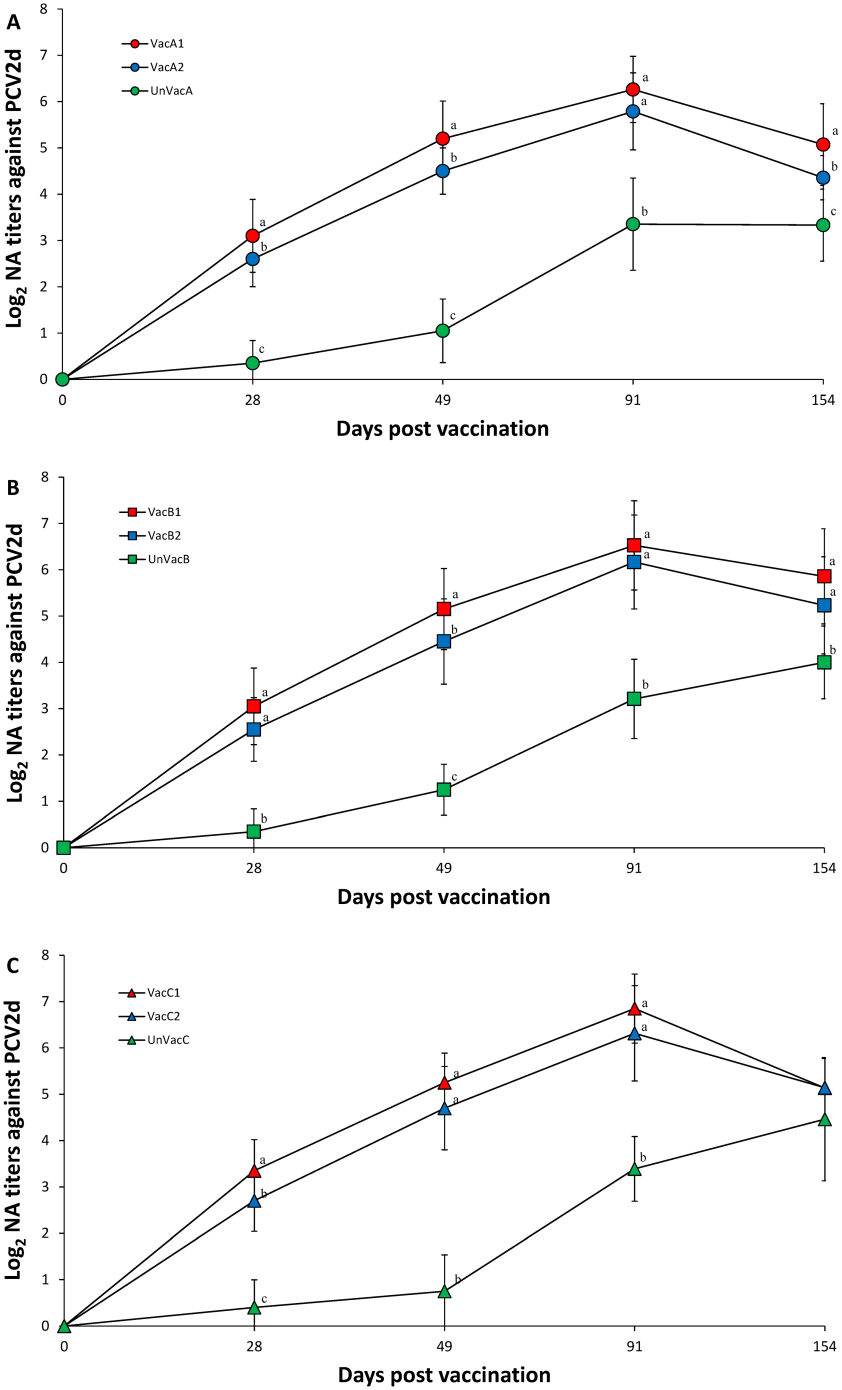
Porcine circovirus type 2d (PCV2d) neutralizing antibodies. **(A)** Mean values of the PCV2d neutralizing antibodies in the farm A. **(B)** Mean values of the PCV2d neutralizing antibodies in the farm B. **(C)** Mean values of the PCV2d neutralizing antibodies in the farm C. Variation is expressed as the standard deviation. Different letters within a sampling point mean statistically significant differences (*p* < 0.05).

Within farm A, the two vaccinated groups (VacA1 and VacA2) had significantly (*p* < 0.05) higher neutralizing antibody titers against PCV2d compared to the unvaccinated group (UnVacA) at 28, 49, 91, and 154 dpv. The all-in-one-vaccinated group (VacA1) had significantly (*p* < 0.05) higher neutralizing antibody titers against PCV2d compared to the concurrent-vaccinated group (VacA2) at 28, 49, and 154 dpv (Figure 5A). Within farm B, the two vaccinated groups (VacB1 and VacB2) had significantly (*p* < 0.05) higher neutralizing antibody titers against PCV2d compared to the unvaccinated group (UnVacB) at 28, 49, 91, and 154 dpv. The all-in-one-vaccinated group (VacB1) had significantly (*p* < 0.05) higher neutralizing antibody titers against PCV2d compared to the concurrent-vaccinated group (VacB2) at 49 dpv (Figure 5B). Within farm C, the two vaccinated groups (VacC1 and VacC2) had significantly (*p* < 0.05) higher neutralizing antibody titers against PCV2d compared to the unvaccinated group (UnVacB) at 28, 49, and 91 dpv. The all-in-one-vaccinated group (VacC1) had significantly (*p* < 0.05) higher neutralizing antibody titers against PCV2d compared to the concurrent-vaccinated group (VacC2) at 28 dpv (Figure 5C).

**Figure 6:**
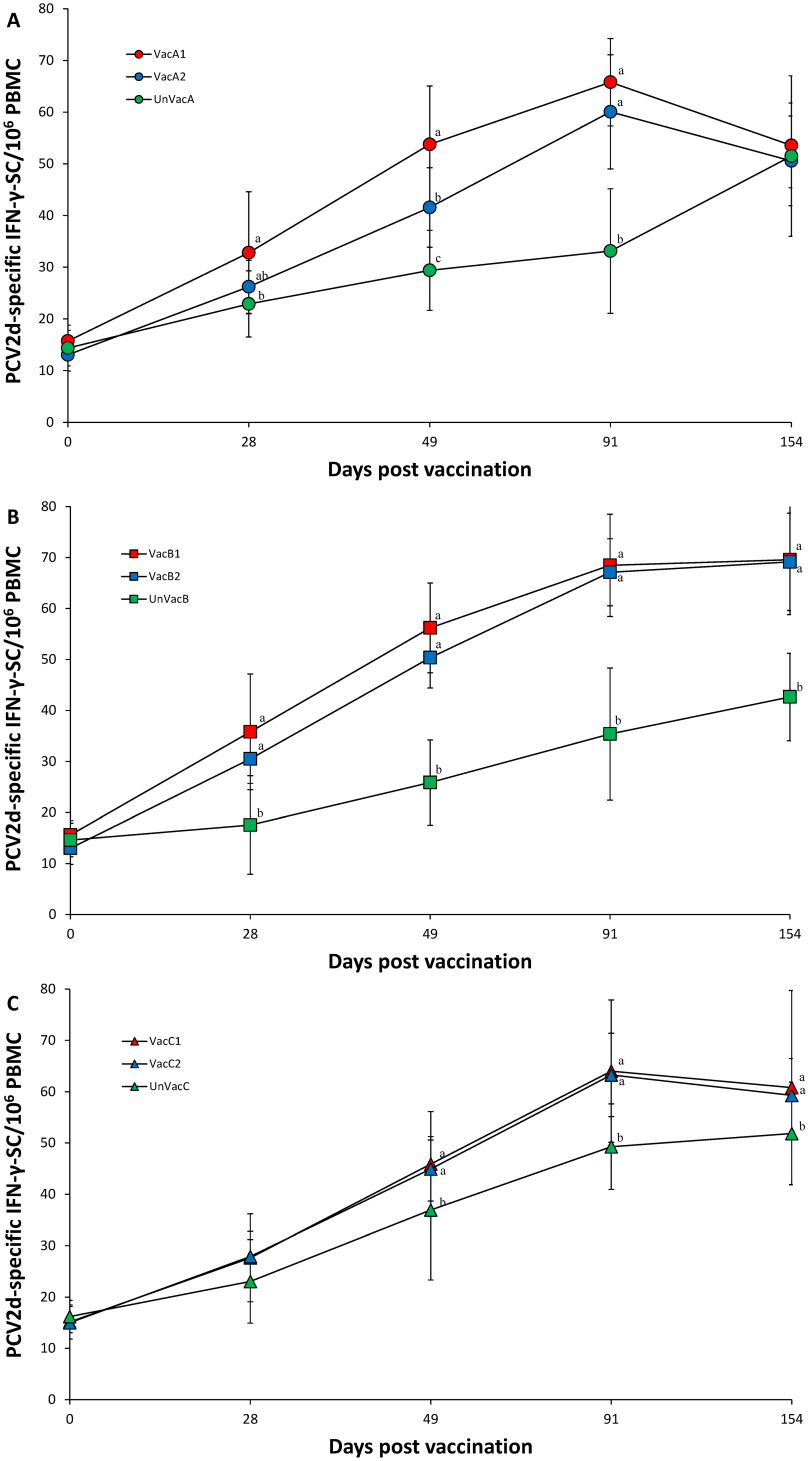
Porcine circovirus type 2d (PCV2d)-specific interferon-γ secreting cells (IFN-γ-SC). **(A)** Mean values of the PCV2d-specific IFN-γ-SC in the farm A. **(B)** Mean values of the PCV2d-specific IFN-γ-SC in the farm B. **(C)** Mean values of the PCV2d-specific IFN-γ-SC in the farm C. Variation is expressed as the standard deviation. Different letters within a sampling point mean statistically significant differences (*p* < 0.05).

Within farm A, vaccination of two groups (VacA1 and VacA2) yielded significantly (*p* < 0.05) higher numbers of PCV2d specific IFN-γ-SC than those of the unvaccinated group (UnVacA) at 28 (except for VacA2), 49, and 91 dpv. The all-in-one-vaccinated group (VacA1) had significantly (*p* < 0.05) higher numbers of PCV2d specific IFN-γ-SC at 49 dpv compared to the concurrent-vaccinated group (VacA2) (Figure 6A). Within farm B, the two vaccinated groups (VacB1 and VacB2) yielded significantly (*p* < 0.05) higher IFN-γ-SC against PCV2d than those of the unvaccinated group (UnVacB) at 28, 49, 91, and 154 dpv (Figure 6B). Within farm C, the two vaccinated groups (VacC1 and VacC2) yielded significantly (*p* < 0.05) higher IFN-γ-SC against PCV2d than those of the unvaccinated group (UnVacB) at 49, 91, and 154 dpv (Figure 6C).

### Immune responses against *Mycoplasma hyopneumoniae*

**Figure 7:**
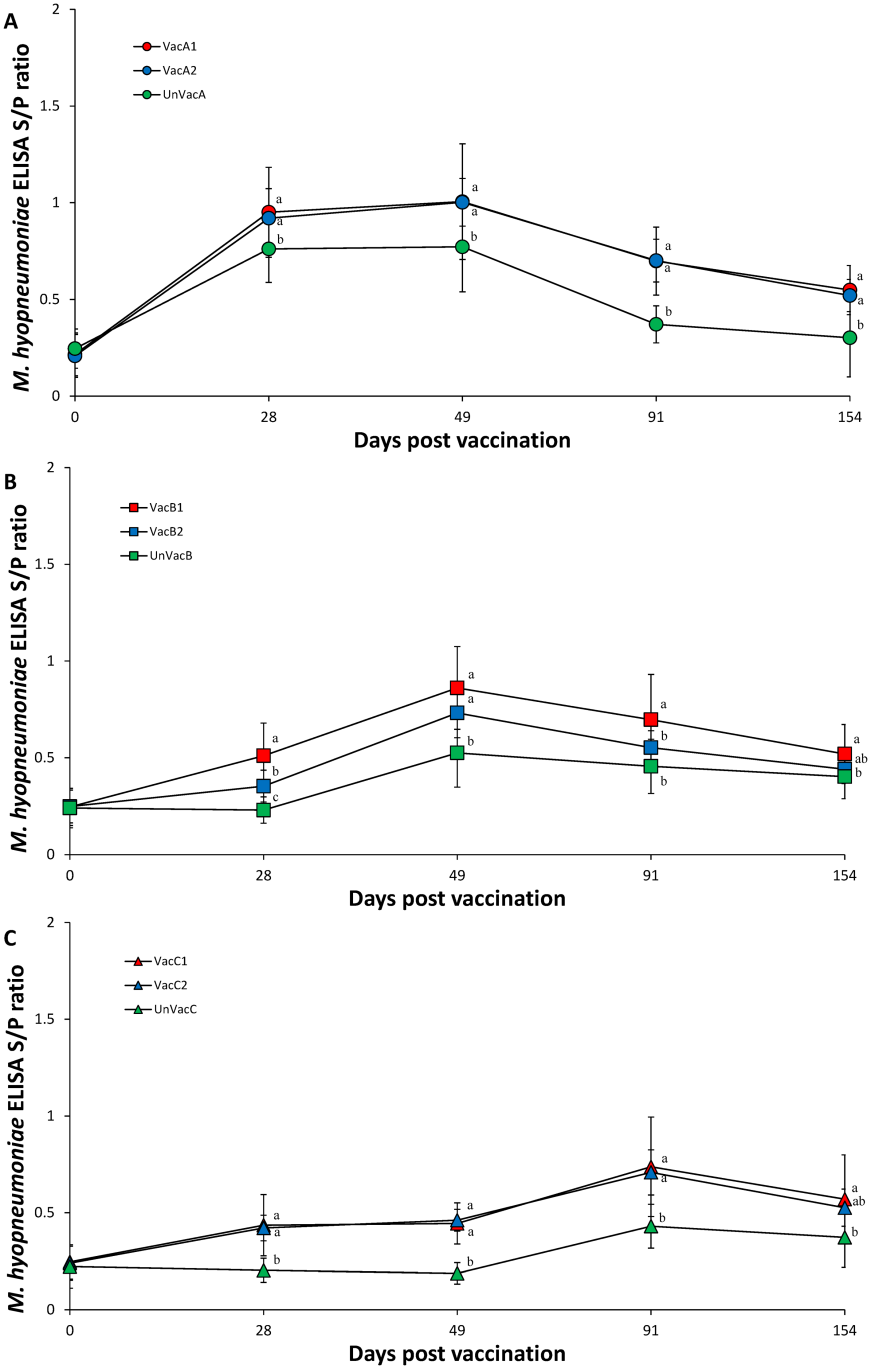
*Mycoplasma hyopneumoniae* antibodies. **(A)** Mean values of the ELISA *M. hyopneumoniae* antibodies in the farm A. **(B)** Mean values of the ELISA *M. hyopneumoniae* antibodies in the farm B. **(C)** Mean values of the ELISA *M. hyopneumoniae* antibodies in the farm C. Variation is expressed as the standard deviation. Different letters within a sampling point mean statistically significant differences (*p* < 0.05).

The two vaccinated groups within farms A and C had significantly (*p* < 0.05) higher *M. hyopneumoniae* S/P ratios than those of their corresponding unvaccinated group at 28, 49, 91, and 154 (except for VacC2) dpv (Figures 7A,C). Within farm B, the all-in-one-vaccinated group (VacB1) had significantly (*p* < 0.05) higher *M. hyopneumoniae* S/P ratios than the unvaccinated group (UnVacB) at 28, 49, 91, and 154 dpv. The all-in-one-vaccinated group (VacB1) had a significantly (*p* < 0.05) higher *M. hyopneumoniae* S/P ratios than the concurrent-vaccinated group (VacB2) at 28 and 91 dpv. The concurrent-vaccinated group (VacB2) had significantly (*p* < 0.05) higher *M. hyopneumoniae* S/P ratios than the unvaccinated group (UnVacB) at 28 and 49 dpv (Figure 7B).

**Figure 8:**
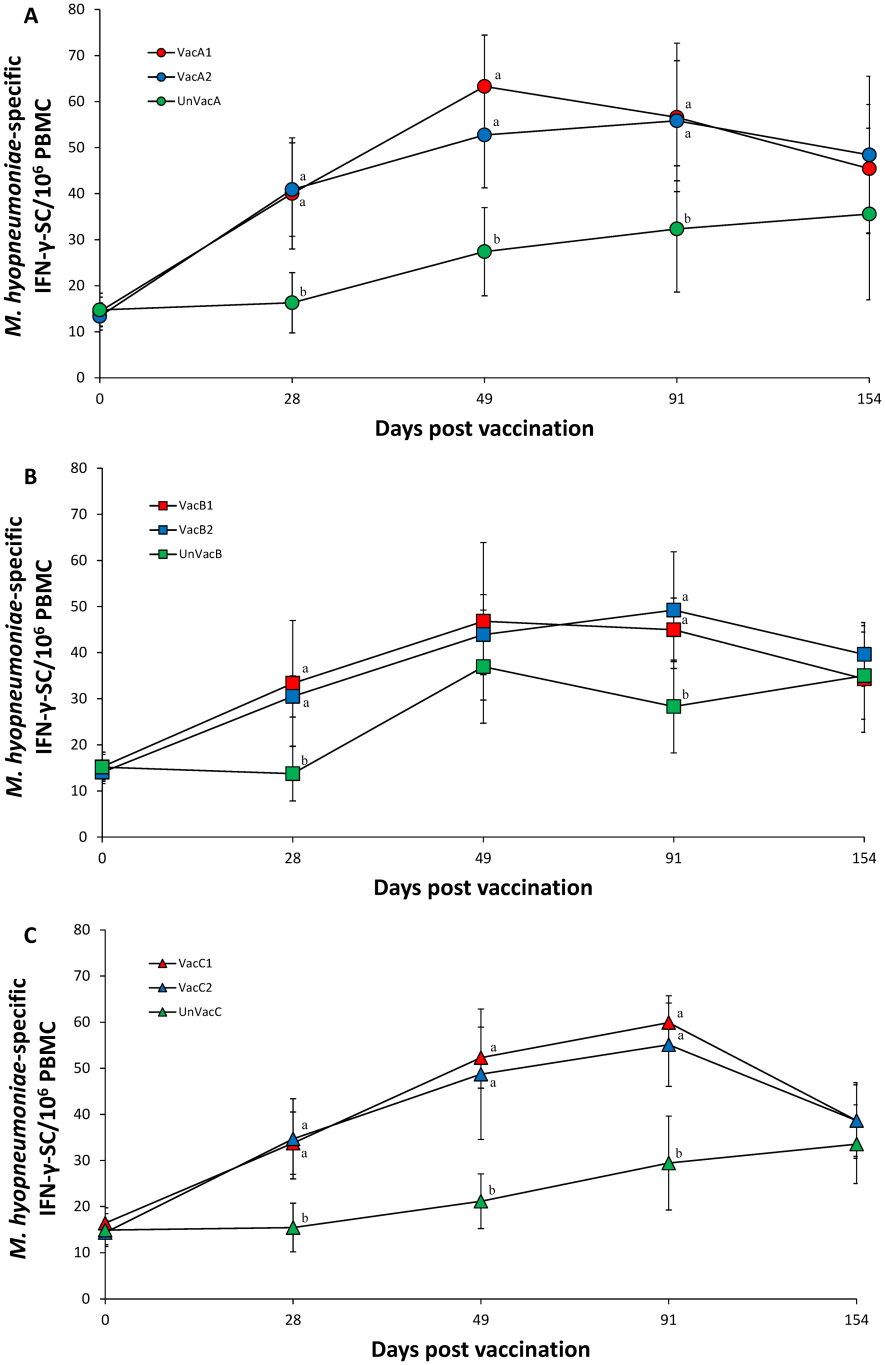
*Mycoplasma hyopneumoniae*-specific interferon-γ secreting cells (IFN-γ-SC). **(A)** Mean values of the *M. hyopneumoniae*-specific IFN-γ-SC in the farm A. **(B)** Mean values of the *M. hyopneumoniae*-specific IFN-γ-SC in the farm B. **(C)** Mean values of the *M. hyopneumoniae*-specific IFN-γ-SC in the farm C. Variation is expressed as the standard deviation. Different letters within a sampling point mean statistically significant differences (*p* < 0.05).

Vaccination of two groups at each farm (A, B, and C) resulted in significantly (*p* < 0.05) higher numbers of *M. hyopneumoniae* specific IFN-γ-SC compared to the corresponding unvaccinated group at 28, 49 (except for farm B), 91 dpv. A significant difference in the number of *M. hyopneumoniae* specific IFN-γ-SC was not found among the three groups (two vaccinated and one unvaccinated) at 49 dpv on farm B (Figures 8A–C).

### Pathology

**Figure 9:**
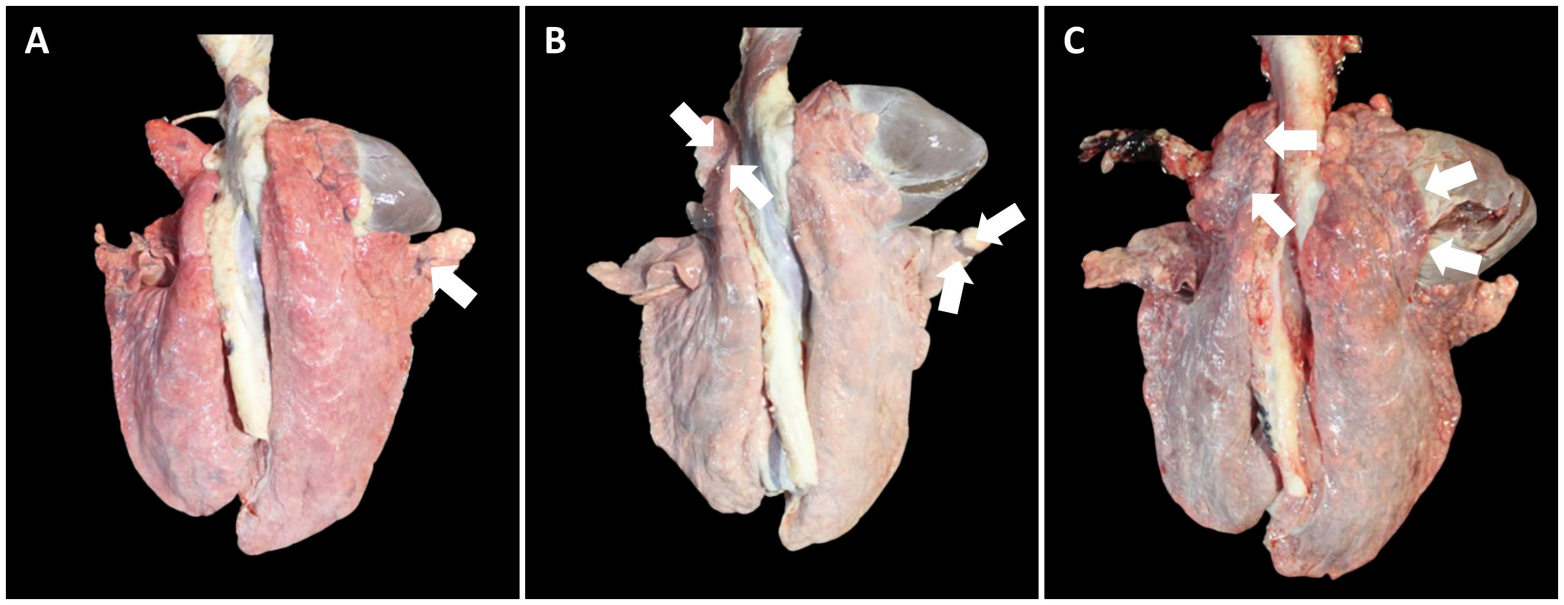
Macroscopic lung lesions in pigs from farm B at 91 dpv. **(A)** Minimal well-demarcated areas of dark-red to purple firm parenchyma (arrow) was observed in pigs from VacB1. **(B)** Moderate well-demarcated areas of dark-red to purple firm parenchyma (arrows) was observed in pigs from VacB2. **(C)** Severe well-demarcated areas of dark-red to purple firm parenchyma (arrows) was observed in pigs from UnVacB.

**Figure 10:**
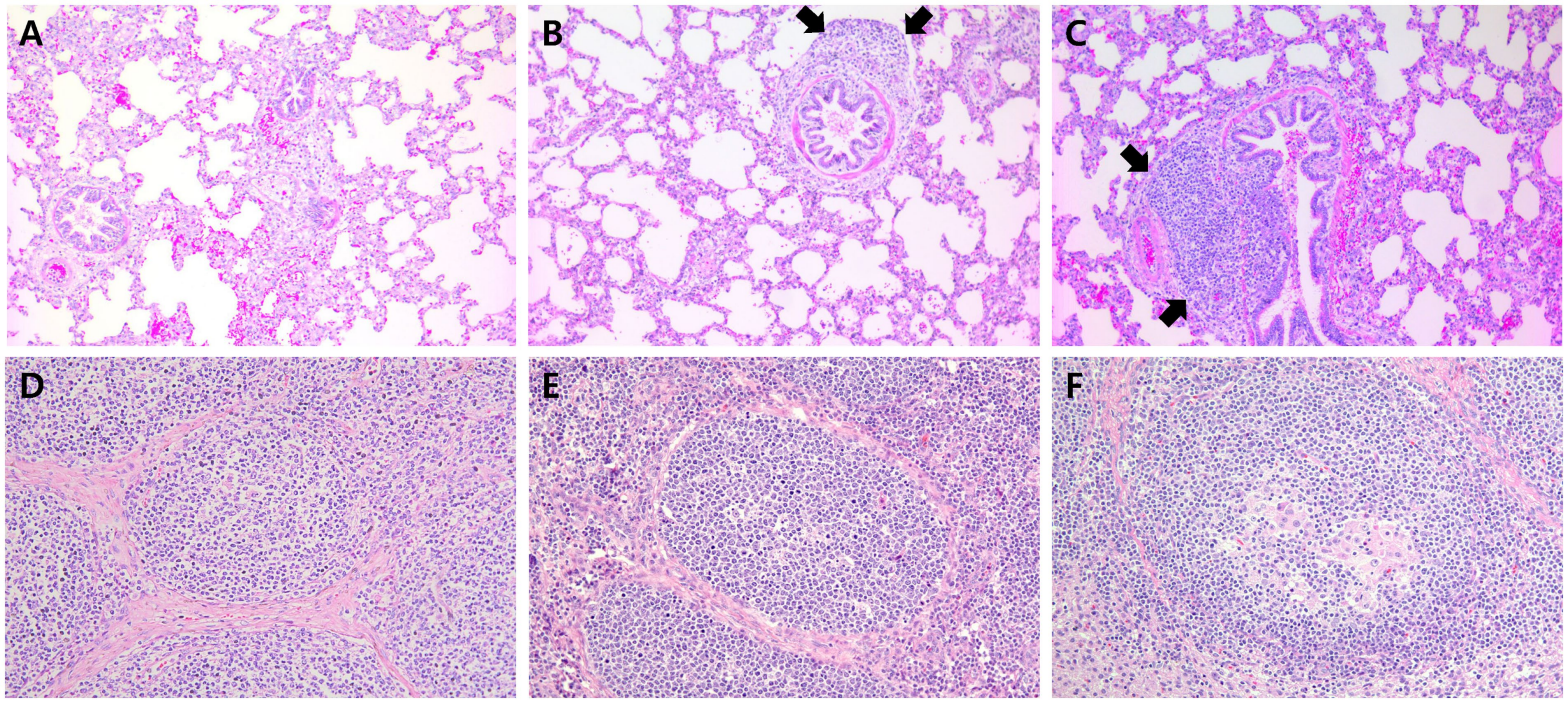
Histopathology of lung in pigs from farm C and lymph node in pigs from farm A at 91 dpv. **(A)** Minimal peribronchiolar lymphoid hyperplasia was observed in pigs from VacC1. **(B)** Moderate peribronchiolar lymphoid hyperplasia (arrows) was observed in pigs from VacC2. **(C)** Severe peribronchiolar lymphoid hyperplasia (arrows) was observed in pigs from VacC2 at 91 dpv. **(D)** Minimal lymphoid depletion was observed in pigs from VacA1. **(E)** Minimal lymphoid depletion was observed in pigs from VacA2. **(F)** Severe diffuse lymphoid depletion and histiocytic replacement in pigs from UnVacA.

The macroscopic lung lesions, characterized by well-demarcated areas of dark-red to purple firm parenchyma, were observed in farms A, B (Figures 9A–C), and C at 91 and 154 dpv. The all-in-one-vaccinated group within farms A, B, and C significantly (*p* < 0.05) lowered the severity of macroscopic pulmonary lesions compared to the scores of the unvaccinated group at 91 and 154 dpv. The microscopic lung lesions, characterized by peribronchiolar lymphoid tissue hyperplasia, were observed in farms A, B, and C (Figures 10A–C) at 91 and 154 dpv. The concurrent-vaccinated group within farms A and C significantly (*p* < 0.05) lowered the severity of macroscopic pulmonary lesions compared to the scores of the unvaccinated group at 91 and 154 (except for farm A) dpv (Table 1). The concurrent-vaccinated group within farms A and C significantly (*p* < 0.05) lowered the severity of microscopic pulmonary lesions compared to the scores of the unvaccinated group at 91 (except for farm C) and 154 dpv (Table 1). Vaccination of both groups within farms A (Figures 10D–F), B, and C significantly (*p* < 0.05) lowered the severity of lymphoid lesions compared to the scores of the unvaccinated group at 91 and 154 dpv (Table 1).

**Table 1:** Pathological outcomes (mean ± standard deviation) in different groups at 91 and 154 days post-vaccination (dpv).

Groups	dpv	Gross lung lesions (%)	Histopathology		PCV2 antigen in lymph node (+cell/0.25 mm^2^)
			Mycoplasmal lung lesions (score 0–6)	Lymph node lesions (score 0–5)	
VacA1	91	7 ± 5.77^b^	0.24 ± 0.17^b^	0.6 ± 0.57^b^	0.93 ± 0.92^b^
	154	6 ± 2.50^b^	0.4 ± 0.24^b^	0.2 ± 0.2^b^	0.33 ± 0.75^b^
VacA2	91	6 ± 7.5^b^	0.32 ± 0.30^b^	0.64 ± 0.71^b^	1.2 ± 1.26^b^
	154	11 ± 7.5^ab^	1 ± 0.47^b^	1.2 ± 0.69^b^	2.8 ± 1.61^b^
UnVacA	91	29 ± 14.93^a^	1.56 ± 0.83^a^	2.72 ± 0.27^a^	4.67 ± 1.7^a^
	154	27 ± 15.55^a^	1.92 ± 0.56^a^	1.96 ± 0.92^a^	10.27 ± 5.84^a^
VacB1	91	6 ± 2.5^b^	0.4 ± 0.32^b^	0.6 ± 0.37^b^	1.6 ± 0.6^b^
	154	5 ± 2.5^b^	0.4 ± 0.2^b^	0.56 ± 0.17^b^	1.4 ± 0.98^b^
VacB2	91	13 ± 9.13^ab^	0.72 ± 0.87^ab^	0.56 ± 0.67^b^	1.47 ± 1.54^b^
	154	11 ± 4.79^ab^	0.92 ± 0.61^b^	1.12 ± 0.36^b^	2.8 ± 0.96^b^
UnVacB	91	30 ± 21.6^a^	1.76 ± 0.71^a^	2.24 ± 1.34^a^	4.8 ± 2.81^a^
	154	29 ± 23.23^b^	2 ± 1.33^a^	2.08 ± 0.61^a^	7.6 ± 3.71^a^
VacC1	91	12 ± 14.43^b^	0.32 ± 0.27^b^	0.64 ± 0.38^b^	1 ± 1^b^
	154	9 ± 4.79^b^	0.32 ± 0.23^b^	0.64 ± 0.22^b^	1 ± 0.53^b^
VacC2	91	11 ± 13.15^b^	0.56 ± 0.62^ab^	0.68 ± 0.64^b^	0.93 ± 2.09^b^
	154	11 ± 7.5^b^	0.76 ± 0.62^b^	1.12 ± 0.56^b^	2.93 ± 1.26^b^
UnVacC	91	39 ± 10.31^a^	1.64 ± 1.07^a^	2.84 ± 0.26^a^	3.93 ± 1.85^a^
	154	39 ± 12.5^a^	1.76 ± 0.78^a^	2.48 ± 0.77^a^	9.8 ± 5.24^a^

Different letters mean statistically significant differences (*p* < 0.05) within the same farm at 91 and 154 days post-vaccination (dpv). The percentage of gross lung lesions and the quantity of PCV2 antigen-positive cells in lymph nodes were assessed for normality using the Shapiro–Wilk test. Normally distributed data were analyzed using one-way ANOVA for comparisons among groups at each time point (dpv 91 and 154), followed by pairwise comparisons with Tukey’s adjustment. Non-normally distributed data, including histopathology scores (mycoplasmal lung lesion score and lymph node lesion score), were analyzed using the Kruskal–Wallis test, with subsequent pairwise comparisons performed using the Mann-Whitney *U* test.

The all-in-one-vaccinated group within farms A, B, and C significantly (*p* < 0.05) lowered the number of lymphoid PCV2 antigen-positive cells from lymph nodes compared to the scores of the unvaccinated group at 91 and 154 dpv. The concurrent-vaccinated group within farms A, B, and C significantly (*p* < 0.05) lowered the number of lymphoid PCV2 antigen-positive cells from lymph nodes compared to the scores of the unvaccinated group at 91 and 154 (except for farm A) dpv (Table 1).

## Discussion

The intradermal vaccines tested in the present field trials were found efficacious against PCV2 and *M. hyopneumoniae* infection. Similarly, intradermal simultaneous two-separated vaccination with monovalent PCV2 and *M. hyopneumoniae* vaccines evaluated in the present study were as effective in herds as in the previous field study (10). Intradermal vaccination improved the production performance in herds suffering from PCV2 infection and enzootic pneumonia. Although a statistical difference was not calculated between the two vaccinated groups about growth improvement, a numerical difference (most likely due to the small animal sample size per group) was noted. Key production parameters such as body weight and ADWG (where a numerical difference was observed), would have resulted in a positive economic impact had the entire herd received the vaccine. Vaccination also benefits pigs indirectly, as observed in these field trials, by reducing viral shedding. Of note, a direct comparison of the two types of intradermal vaccines found that the all-in-one vaccine resulted in a significantly greater reduction of fecal and nasal shedding of PCV2d and mycoplasma nasal shedding compared to the concurrent vaccine. These clinical data indicate that the all-in-one vaccination reduced the subsequent infectious pressure and the positive effects would only increase if all study pigs had been vaccinated in the same manner.

Intradermal vaccination evoked systemic humoral and cell-mediated immune responses to *M. hyopneumoniae*. In particular, the cell-mediated immune response measured by the number of IFN-γ-SC correlated well with pig protection against *M. hyopneumoniae* (26–28). The development of IFN-γ-SC by an intradermal vaccine resulted in the reduction of *M. hyopneumoniae* loads in the larynx and mycoplasmal lung lesion severity. Intradermal vaccination is, therefore, able to elicit sufficient levels of cell-mediated protective immunity to protect pigs against *M. hyopneumoniae* infection.

Intradermal vaccination can also stimulate systemic humoral and cell-mediated immune responses to PCV2d. In particular, it has been reported that neutralizing antibodies and IFN-γ-SC against specific PCV2 was well correlated with the reduction of PCV2 viremia (29, 30). In the present study, vaccination of pigs with the intradermally administered vaccine elicited neutralizing anti-PCV2d antibody and PCV2d-specific IFN-γ-SC, which reduced the levels of PCV2d blood viral loads. The reduction of PCV2 viremia is critical in reducing lymphoid lesions (27, 28). Therefore, intradermal vaccination elicited sufficient levels of humoral and cell-mediated protective immunity to protect pigs against PCV2d infection. Nevertheless, there is a slight difference in the immunokinetics between the all-in-one and concurrent vaccine. Essentially, the all-in-one vaccine elicits significantly higher neutralizing antibody titers against PCV2d (in two farms) and a higher frequency of PCV2d-specific IFN-γ-SC than those that received the concurrent vaccine at 4 weeks post-vaccination (in one farm). These results indicate that the intradermal all-in-one vaccine was more efficacious than the intradermal concurrent vaccine in protecting pigs against early PCV2d infection.

The all-in-one vaccine was explicitly developed for intradermal administration using a needle free and intradermal injector. The needle-free device allows a dose volume of 0.2 mL, one-tenth of the volume typically administered for a commercially available intramuscular bivalent PCV2 and *M. hyopneumoniae* vaccine. All-in-one and concurrent intradermal vaccines are safer for pigs since they do not cause postvaccinal adverse local (i.e., redness, swelling, and abscess) and systemic (i.e., diarrhea, anaphylactic shock, and lethargy) reactions (data not shown). Noteworthy, the intradermal all-in-one vaccine is a completely ready-to-use vaccine and, therefore, more convenient to use than the intradermal mixed vaccine for farm workers.

The all-in-one intradermal vaccine evaluated is based on the recombinant *M. hyopneumoniae* strain with an embedded/integrated PCV2 capsid protein thereof as the single active substance that provides cross-protection against PCV2d in herds suffering from subclinical PCV2d infection. The results of the present field trials are consistent with the previous experimental study, where the same all-in-one intradermal vaccine also conferred the cross-protection of pigs against a dual PCV2d and *M. hyopneumoniae* challenge under experimental conditions (11). A genetic difference exists between PCV2a (what most conventional injectable vaccines are made with) and PCV2d (the most prevalent PCV2 genotype in worldwide circulation that causes infections). Despite this genetic difference, PCV2a-based vaccines are able to cross-protect against PCV2d in previous studies (14, 31). This cross-protection of PCV2d by a PCV2a-based vaccine is clinically important to the Asian pork industry, as PCV2d is the most predominant genotype circulating within Asian pig farms (32–35). An intradermal all-in-one vaccine comprising *M. hyopneumoniae* and PCV2 is a valuable tool in protecting pig herds suffering from PCV2d infection and enzootic pneumonia.

## Data Availability

The original contributions presented in the study are included in the article/supplementary material, further inquiries can be directed to the corresponding author.

## Footnotes

^1^ http://imagej.nih.gov/ij/download.html

## References

[1] Chae C. Postweaning multisystemic wasting syndrome: a review of aetiology, diagnosis and pathology. Vet J. (2004) 168:41–9. doi: 10.1016/s1090-0233(03)00182-5, PMID: 15158207

[2] Chae C. A review of porcine circovirus 2-associated syndromes and diseases. Vet J. (2005) 169:326–36. doi: 10.1016/j.tvjl.2004.01.012, PMID: 15848776

[3] Segalés J. Porcine circovirus type 2 (PCV2) infections: clinical signs, pathology and laboratory diagnosis. Virus Res. (2012) 164:10–9. doi: 10.1016/j.virusres.2011.10.007, PMID: 22056845

[4] Maes D Verdonck M Deluyker H de Kruif A. Enzootic pneumonia in pigs. Vet Q. (1996) 18:104–9. doi: 10.1080/01652176.1996.9694628, PMID: 8903144

[5] Pieters M Maes D. Mycoplasmosis In: Zimmerman JJ Karriker LA Ramirez A Schwartz KJ Stevenson GW Zhang J, editors. Diseases of swine. Hoboken, NJ: Wiley-Blackwell (2019). 863–83.

[6] Kim J Chung HK Chae C. Association of porcine circovirus 2 with porcine respiratory disease complex. Vet J. (2003) 166:251–6. doi: 10.1016/s1090-0233(02)00257-5, PMID: 14550736

[7] Hansen MS Pors SE Jensen HE Bille-Hansen V Bisgaard M Flachs EM. An investigation of the pathology and pathogens associated with porcine respiratory disease complex in Denmark. J Comp Pathol. (2010) 143:120–31. doi: 10.1016/j.jcpa.2010.01.012, PMID: 20181357 PMC7094415

[8] Ferrari L Borghetti P Gozio S De Angelis E Ballotta L Smeets J. Evaluation of the immune response induced by intradermal vaccination by using a needle-less system in comparison with the intramuscular route in conventional pigs. Res Vet Sci. (2011) 90:64–71. doi: 10.1016/j.rvsc.2010.04.026, PMID: 20546827

[9] Tassis PD Papatsiros VG Nell T Maes D Alexopoulos C Kyriakis SC. Clinical evaluation of intradermal vaccination against porcine enzootic pneumonia (*Mycoplasma hyopneumoniae*). Vet Rec. (2012) 170:261. doi: 10.1136/vr.100239, PMID: 22262700

[10] Sno M Cox E Holtslag H Nell T Pel S Segers R. Efficacy and safety of a new intradermal PCV2 vaccine in pigs. Trials Vaccinol. (2016) 5:24–31. doi: 10.1016/j.trivac.2016.01.002

[11] Suh J Oh T Chae C. An evaluation of intradermal all-in-one vaccine based on an inactivated recombinant *Mycoplasma hyopneumoniae* strain expression porcine circovirus (PCV2) capsid protein against Korean strains of PCV2d and *M. hyopneumoniae* challenge. Comp Immunol Microbiol Infect Dis. (2022) 90-91:101911. doi: 10.1016/j.cimid.2022.101911, PMID: 36410070

[12] Beaver BV Reed W Leary S McKiernan B Bain F Schultz R. 2000 report of the AVMA panel on euthanasia. J Am Vet Med Assoc. (2001) 218:669–96. doi: 10.2460/javma.2001.218.669, PMID: 11280396

[13] Park KW Cho H Suh J Oh T Park Y Park S. Field evaluation of novel plant-derived porcine circovirus type 2 vaccine related to subclinical infection. Vet Med Sci. (2023) 9:2703–10. doi: 10.1002/vms3.1256, PMID: 37665771 PMC10650242

[14] Park KH Oh T Yang S Cho H Kang I Chae C. Evaluation of a porcine circovirus type 2a (PCV2a) vaccine efficacy against experimental PCV2a, PCV2b, and PCV2d challenge. Vet Microbiol. (2019) 231:87–92. doi: 10.1016/j.vetmic.2019.03.002, PMID: 30955830

[15] Jeong J Park C Choi K Chae C. Comparison of three commercial one-dose porcine circovirus type 2 (PCV2) vaccines in a herd with concurrent circulation of PCV2b and mutant PCV2b. Vet Microbiol. (2015) 177:43–52. doi: 10.1016/j.vetmic.2015.02.027, PMID: 25790733

[16] Dubosson CR Conzelmann C Miserez R Boerlin P Frey J Zimmermann W. Development of two real-time PCR assays for the detection of *Mycoplasma hyopneumoniae* in clinical samples. Vet Microbiol. (2004) 102:55–65. doi: 10.1016/j.vetmic.2004.05.007, PMID: 15288927

[17] Kurth KT Hsu T Snook ER Thacker EL Thacker BJ Minion FC. Use of a *Mycoplasma hyopneumoniae* nested polymerase chain reaction test to determine the optimal sampling sites in swine. J Vet Diagn Invest. (2002) 14:463–9. doi: 10.1177/104063870201400603, PMID: 12423027

[18] Fort M Olvera A Sibila M Segalés J Mateu E. Detection of neutralizing antibodies in postweaning multisystemic wasting syndrome (PMWS)-affected and non-PMWS-affected pigs. Vet Microbiol. (2007) 125:244–55. doi: 10.1016/j.vetmic.2007.06.004, PMID: 17611048

[19] Pogranichnyy RM Yoon KJ Harms PA Swenson SL Zimmerman JJ Sorden SD. Characterization of immune response of young pigs to porcine circovirus type 2 infection. Viral Immunol. (2000) 13:143–53. doi: 10.1089/vim.2000.13.143, PMID: 10892995

[20] Jeong J Kang I Kim S Park KH Park C Chae C. Comparison of 3 vaccination strategies against porcine reproductive and respiratory syndrome virus, *Mycoplasma hyopneumoniae*, and porcine circovirus type 2 on 3 pathogen challenge model. Can J Vet Res. (2018) 82:39–47. PMID: 29382967 PMC5764041

[21] Halbur PG Paul PS Frey ML Landgraf J Eernisse K Meng XJ. Comparison of the pathogenicity of two US porcine reproductive and respiratory syndrome virus isolates with that of the Lelystad virus. Vet Pathol. (1995) 32:648–60. doi: 10.1177/030098589503200606, PMID: 8592800

[22] Opriessnig T Thacker EL Yu S Fenaux M Meng XJ Halbur PG. Experimental reproduction of postweaning multisystemic wasting syndrome in pigs by dual infection with *Mycoplasma hyopneumoniae* and porcine circovirus type 2. Vet Pathol. (2004) 41:624–40. doi: 10.1354/vp.41-6-624 15557072

[23] Kim J Chae C. Expression of monocyte chemoattractant protein-1 and macrophage inflammatory protein-1 in porcine circovirus 2-induced granulomatous inflammation. J Comp Pathol. (2004) 131:121–6. doi: 10.1016/j.jcpa.2004.02.001, PMID: 15276851

[24] Park C Oh Y Seo HW Han K Chae C. Comparative effects of vaccination against porcine circovirus type 2 (PCV2) and porcine reproductive and respiratory syndrome virus (PRRSV) in a PCV2-PRRSV challenge model. Clin Vaccine Immunol. (2013) 20:369–76. doi: 10.1128/cvi.00497-12, PMID: 23302743 PMC3592349

[25] Kim J Choi C Chae C. Pathogenesis of postweaning multisystemic wasting syndrome reproduced by co-infection with Korean isolates of porcine circovirus 2 and porcine parvovirus. J Comp Pathol. (2003) 128:52–9. doi: 10.1053/jcpa.2002.0605, PMID: 12531687

[26] Thacker EL Thacker BJ Kuhn M Hawkins PA Waters WR. Evaluation of local and systemic immune responses induced by intramuscular injection of a *Mycoplasma hyopneumoniae* bacterin to pigs. Am J Vet Res. (2000) 61:1384–9. doi: 10.2460/ajvr.2000.61.1384, PMID: 11108184

[27] Yang S Oh T Park KH Cho H Suh J Chae C. Experimental efficacy of a trivalent vaccine containing porcine circovirus types 2a/b (PCV2a/b) and *Mycoplasma hyopneumoniae* against PCV2d and *M. hyopneumoniae* challenge. Vet Microbiol. (2021) 258:109100. doi: 10.1016/j.vetmic.2021.109100, PMID: 33984792

[28] Ham S Suh J Oh T Kin C Seo BJ Chae C. Efficacy of a novel bivalent vaccine containing porcine circovirus type 2d and *Mycoplasma hyopneumoniae* against a dual PCV2d and *Mycoplasma hyopneumoniae* challenge. Front Vet Sci. (2023) 10:1176091. doi: 10.3389/fvets.2023.1176091, PMID: 37565086 PMC10410152

[29] Meerts P Van-Gucht S Cox E Vandebosch A Nauwynck HJ. Correlation between type of adaptive immune response porcine circovirus type 2 and level of virus replication. Viral Immunol. (2005) 18:333–41. doi: 10.1089/vim.2005.18.333, PMID: 16035945

[30] Meerts P Misinzo G Lefebvre D Nielsen J Botner A Kristensen CS. Correlation between the presence of neutralizing antibodies against porcine circovirus 2 (PCV2) and protection against disease. BMC Vet Res. (2006) 2:6–16. doi: 10.1186/1746-6148-2-6, PMID: 16445856 PMC1386657

[31] Oh T Suh J Cho H Min K Choi BH Chae C. Efficacy test of a plant-based porcine circovirus type 2 (PCV2) virus-like particle vaccine against four PCV2 genotypes (2a, 2b, 2d, and 2e) in pigs. Vet Microbiol. (2022) 272:109512. doi: 10.1016/j.vetmic.2022.109512, PMID: 35853407

[32] Park KH Chae C. The prevalence of porcine circovirus type 2e (PCV2e) in Korean slaughter pig lymph nodes when compared with other PCV2 genotypes. Transbound Emerg Dis. (2021) 68:3043–7. doi: 10.1111/tbed.13975, PMID: 33406315

[33] Thangthamniyom N Sangthong P Poolperm P Thanantong N Boonsoongnern A Hansoongnern P. Genetic diversity of porcine circovirus type 2 (PCV2) in Thailand during 2009–2015. Vet Microbiol. (2017) 208:239–46. doi: 10.1016/j.vetmic.2017.08.006, PMID: 28888644

[34] Tsai GT Lin YC Lin WH Lin JH Chiou MT Liu HF. Phylogeographic and genetic characterization of porcine circovirus type 2 in Taiwan from 2001–2017. Sci Rep. (2019) 9:10782. doi: 10.1038/s41598-019-47209-1, PMID: 31346205 PMC6658515

[35] Yang S Yin S Shang Y Liu B Yuan L Zafar Khan MU. Phylogenetic and genetic variation analyses of porcine circovirus type 2 isolated from China. Transbound Emerg Dis. (2018) 65:e383–92. doi: 10.1111/tbed.12768, PMID: 29150903

